# Wound to the Brain

**Published:** 1879-10-20

**Authors:** Thos. O. Summers

**Affiliations:** Nashville, Tennessee


### *A REMARKABLE CASE OF WOUND TO THE BRAIN.*

BY THOS. O. SUMMERS, M.D., OF NASHVILLE, TENN.

There is perhaps no literature so uninviting to a practitioner who relies upon his Journal for keeping *en rapport* with the advances of the day, than reports of cases. And yet there are some cases which arise in the daily practice of the surgeon especially, which throw great light upon modes of procedure involving annoying perplexity, since the general works upon surgery, from the very nature of the case, cannot guide the operator in details, on which the success of an operation frequently hangs. The report which I propose now to submit, offers one of that character, and may furnish guidance in others likely at any time to occur.

One very warm evening in October, 1876, a messenger came into my office from the Coroner, requesting me to come at once to make an autopsy upon a man who "had been killed in a difficulty in a saloon near by."

On arriving at the place I found the jury just about organizing, and the subject lying upon the floor in a pool of blood and brain matter, apparently lifeless. Upon examination I found the pulse still beating, and signs of reaction, which of course called the inquest to a halt, and I suggested to the worthy Coroner, more jocularly than otherwise, that I might yet cheat him out of his fee, and set immediately to work. With a little warm water I syringed out the wound, the opening of which I found just one quarter of an inch in front of the meatus auditorius externus. Having thus cleared out the track of the wound, I let a flexible filiform bougie drop into it, and found by delicate manipulation that the ball had ranged slightly forward along the upper surface of the petrous portion of the temporal bone of the left side—the side of entrance—passing just over the Casserian ganglion, breaking off the left posterior clinoid process of the sphenoid, and finally becoming lost upon the right side, just in front of the petrous portion of the right temporal bone. I carefully removed all spicula of bone and the debris of soft tissues destroyed in the track of th · bullet, applied ice to the wound, and carried the patient to his home in Edgefield, *a mile across the river*, in an ambulance. On visiting him in the morning I found him utterly unconscious, breathing heavily with slight stertor. There was considerable singultus, and *subsultus tendinum* in the flexors of the left forearm. Finding him able to swallow I administered at once two drachms of bromide of potassium and proceeded to examine the wound more thoroughly. As far as I could gather from the relation of the wounded parts, the middle meningeal was the only artery of any importance which had been cut, and that was closed at both extremities by a clot. I made no attempt of course to ligate, but left it to be plugged by coagula, relying upon emptying the brain of its blood by the action of the bromida and by ice applied externally. The ball being a very small one—Smith & Wesson's smallest—I could not introduce the smallest speculum, and so trusted entirely to the surgical anatomy of the parts, to determine what lesions I had to deal with. Coming to the conclusion as above stated, I now ordered three bladders filled with ice, to be applied to both temples and to the *corona capitis*. These were not removed for two weeks, except to be replenished with ice. I removed the jagged edges of the bone around the wound, and left him with two drachms of bromide to be taken three times a day and at night the same quantity, with forty grains of hydrate of chloral added. I administered on the third day ten grains of calomel, and allowed him nothing but broth diet. He gradually improved under this treatment, until on the fifteenth day he was able to sit up. There was an entire loss of memory concerning all events transpiring after the difficulty, and though I visited him twice a day, he never recalled my face, though conversing rationally with me concerning his symptoms at every visit. His immediate family he seemed to recognize as everything with which he was familiar before the difficulty was perfectly clear. This is a psychological problem as yet unsolved by me.

Four weeks after the wound was made, the patient was out and the memory soon began to return. The neuralgia, however, was intense, and I feared that this might finally destroy entirely the functional activity of the brain. Some two months after he returned to work, with but little impairment of his cerebral functions. In a short while he showed signs of advancing epilepsy, which was, however, gradually controlled by heavy doses of the bromide of potassium, for which I often substituted the bromide of sodium to preserve the integrity of the stom ach. His appetite was ravenous, but beyond this, and the occasional epileptic attacks, he was just the same mentally, as before the wounding, though he had lost about two tablespoonfuls of cerebral matter. His speech at first was slightly affected, and swallowing difficult. This, however, was relieved in the course of time.

Two years passed away, and during my absence at the epidemic of 1878, he was engaged in another encounter, and received a ball in Scarpa's triangle, I believe, for which he was successfully treated by Prof. W. T. Briggs.

I should further have stated that during the two years intervening between the two difficulties, I had placed him under treatment for secondary syphilis, giving him daily for several months the enormous quantity of one hundred grains of the iodide of potassium in fl. ext. of sarsaparilla, until all symptoms had disappeared. Four weeks ago at this writing (July 1st) I determined to open the wound to determine, if possible, the cause of the epileptic fits, which by their frequency and violence had incapacitated him for labor and rendered his existence a burden to him. He was never for the four months preceding free from pain, which yielded to none of the ordinary remedies. I dared not use opium upon him for fear of producing hyperæmia of the brain.

Assisted by Dr. R. W. Steger, I laid back a flap from over the wound, which we found puffed out with a membranous sac, enveloping pus and a small piece of bone pressing directly upon the brain. All of this was carefully removed, and the former track of the wound explored. We found this perfectly free from obstruction, a canal of connective tissue having formed a protecting covering to the lacerated brain surface of the channel. The trephine and Hay's saw were applied to smooth the projecting edges of bone, and with the elevator and scissors, the depressed callous was extracted. The flap was loosely laid back over the wound and left to heal, which it did with not a single unfavorable symptom—the patient rapidly recovering with no sign of epilepsy, and perfect immunity from the distressing nenralgia under which he had so terribly suffered.

The response of this patient to the remedial means employed was indeed remarkable, and his recovery almost miraculous.

We have several cases recorded in which the brain suffered even a greater extent of lesion than this, but none, I am safe in the assertion, which so closely involved vital structures, and under circumstances more unfavorable to complete recovery. I have given thus the details of this case, as I believe it worthy of record in the history of surgery. —*Nashville Journal of Medicine and Surgery.*

